# Successful Use of the MYNXGRIP Closure Device during Repeated Transbrachial Percutaneous Peripheral Intervention

**DOI:** 10.1155/2015/346506

**Published:** 2015-08-26

**Authors:** Klaus Hertting, Werner Raut

**Affiliations:** Department of Cardiology and Angiology, Krankenhaus Buchholz, 21244 Buchholz in der Nordheide, Germany

## Abstract

The use of closure devices after transbrachial arterial puncture is still controversial. Here we report on a case where the MYNXGRIP (AccessClosure Inc., Santa Clara, CA, USA) could be used successfully in a patient, who underwent percutaneous peripheral arterial intervention twice via transbrachial access.

## 1. Introduction

Percutaneous vascular interventions via the brachial artery (BA) represent a commonly used vascular access. Today, manual compression is the most widely used way to close the arterial puncture [1, 2]. The local complication rate of brachial access route is up to seven percent (older studies report even higher event rates), mostly comprising large hematomas, false aneurysms, thrombotic occlusions, or nerve injuries with subsequent dysfunction of the forearm [1, 2].

Dedicated closure devices in the femoral artery were tested in a series of studies and registries [3, 4]. In a small retrospective study, Mirza et al. reported no significant difference regarding vascular complications after the use of closure devices or manual compression for closure of BA puncture [5].

Here, we report the repeated use of the MYNXGRIP closure device (AccessClosure Inc., Santa Clara, CA, USA) in a patient who required a staged revascularization for bilateral critical limb ischemia. The device uses a biodegradable polyethylene-glycol sealant attaching to the outer layer of the vessel wall. The sealant is administered through the sheath while an inflated balloon inside the vessel provides temporary hemostasis and prevents protrusion of the sealant into the lumen [6]. The patient gave informed consent for the publication.

## 2. Case Presentation

We report on a 69-year-old lady who presented with bilateral critical limb ischemia (Rutherford V) caused by a high grade stenosis of the left and an occlusion of the right common femoral artery (CFA). As the patient had significant comorbidities (mild dementia, liver cirrhosis, and reduced kidney function) and has had surgery of both CFA previously, it was decided to try an interventional revascularization of both CFA. A transbrachial access route with the use of a closure device was considered as appropriate in this situation. Preprocedural ultrasound revealed a left BA without relevant atherosclerosis and a diameter of 3.4 mm. The patient was pretreated with 100 mg aspirin daily.

After puncturing the low brachial artery (using a 21-gauge needle and ultrasound guidance) and administration of 5000 units of heparin, a 90 cm 6F sheath was introduced and angioplasty with stent implantation into the left external iliac and CFA could be performed with good angiographic result. The occlusion of the right CFA was scheduled for another intervention.

**Figure 1:**
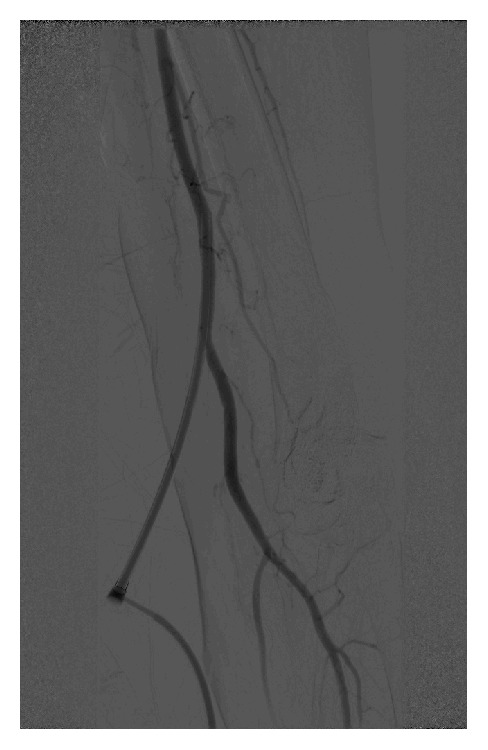
Angiography of the left brachial artery at the end of the first procedure after placement of a 6F-10 cm sheath.

**Figure 2:**
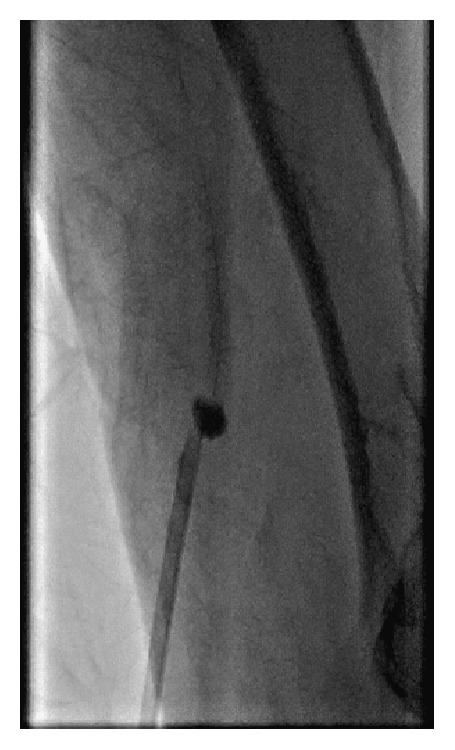
Retrieval of the inner blocking balloon of the MYNXGRIP and of the sheath towards the puncture site. Note the slight shift between the sheath and the blocking balloon indicating appropriate wall contact.

The 90 cm sheath was then exchanged for a 10 cm 6F sheath. Subsequently, an angiography has been performed after intra-arterial application of 200 *μ*g nitroglycerin [Figure 1]. The balloon of the MYNXGRIP was prepared using a mixture of contrast-dye and saline to allow visualization during the placement. The device was inserted into the sheath and the balloon inflated and slowly withdrew towards the puncture site under fluoroscopic surveillance [Figure 2]. After confirming appropriate wall contact the MYNXGRIP sealant was applied according to the instructions for use. Finally, after confirmation of hemostasis, the puncture site was covered by a small dressing avoiding extensive compression of the artery. Radial and ulnar pulse proved to be strongly palpable. After procedure the patient received a loading dose of 600 mg clopidogrel and then 75 mg daily.

**Figure 3:**
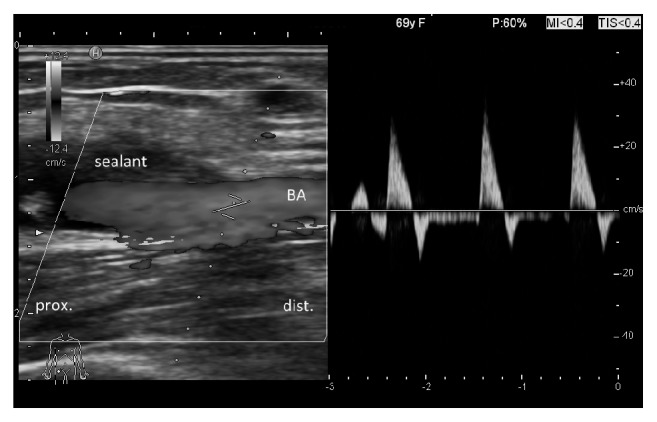
Ultrasound study of the left brachial artery the day after the first procedure (BA, brachial artery).

Duplex ultrasound control the day after the procedure showed an echolucent area at the puncture site, representing the MYNXGRIP sealant and patent brachial, radial, and ulnar arteries [Figure 3].

Two days later, a repeated puncture about 1 cm central to the previous puncture site was performed again under ultrasound guidance. Recanalization and stenting of the right CFA could be performed. Angiography of the BA at the end of the procedure showed a patent BA with preserved flow into the forearm. After exchanging for a 6F 10 cm sheath a MYNXGRIP device could be placed without problems.

Duplex ultrasound the day after the second procedure revealed a mild diffuse subcutaneous hematoma without signs of false aneurysm, av-fistula, dissection, or thrombosis but with regular flow in the BA and into the radial and ulnar arteries. This result could be confirmed after 7 days prior to patients discharge. No relevant clinical impairments of the left arm occurred.

## 3. Discussion

Here we report on the repeated use of the MYNXGRIP closure device in the left brachial artery (BA) for peripheral intervention. To our knowledge, this is the first report of usage in this setting.

Puncturing the BA is somewhat different in comparison to the common femoral artery (CFA). First, the diameter of the adult BA ranges from 3 to 6 mm, whereas the CFA usually provides a larger diameter [7, 8]. Second, the BA is more susceptible for vascular spasm [1]. Third, the amount of subcutaneous tissue is less in the BA than in the CFA area [1]. Fourth, the puncture site of the BA is less well defined than that of the CFA. The range of anatomic variabilities of the BA comprises variable origins of the forearm arteries, variable courses of the BA, a highly variable deep venous system, and so forth [1]. Thus, puncturing the BA sometimes is more difficult and eventually requires more dedicated techniques (e.g., ultrasound guidance).

So far, the use of different closure devices in the BA has been published. The largest series comes from Lupattelli et al., reporting on 159 patients where an Angio-Seal (St. Jude Medical, St. Paul, MN, USA) closure device had been used with high success and low complication-rates, but also smaller registries exist [9, 10]. Of note, in the registry of Lupattelli in 79 of the 238 patients (33%) with brachial access a closure device had not been implanted, mostly because the diameter of the artery appeared too small [9]. Other reports describe the use of different closure devices, such as nitinol clips or suture closures also with high safety and success rates [11, 12].

The main differences of the MYNXGRIP-system are the fact that theoretically it leaves no material (neither permanent nor degradable) inside the vessel lumen and additionally leaves no permanent material in or directly adjacent to the vessel wall, as done by other devices [6]. Nevertheless, some authors report a relevant rate of intraluminal migration of the sealant material (18%) or formation of false aneurysm (11%) [13–15]. Grandhi et al. reported in their analysis on the use of the MYNX device in transfemoral cerebrovascular interventions an association of lower body-mass index and complication rate [16]. Whether the safety and efficacy of the device are comparable to other systems still remains unclear so far [17–19]. However, patient comfort may be higher with the use of the MYNXGRIP compared to the Angio-Seal [14]. Garasic et al. investigated the successful use of the MYNXGRIP device in repeated arterial puncture in a sheep model [20].

Possible problems with the use of the MYNXGRIP-device in the BA are (1) the development of significant spasm precluding the inner balloon to get in appropriate contact with the vessel wall at the puncture site, (2) dislodgement of the MYNX sealant into the arterial lumen causing a thrombotic occlusion, (3) venous thrombosis due to placement of the MYNX sealant after accidentally puncturing the artery through an adjacent vein, (4) protrusion of MYNX sealant above the level of epidermis because of a shorter puncturing channel in comparison to CFA puncture, (5) secondary infections, and (6) failure of the device to achieve adequate hemostasis.

To avoid these pitfalls we recommend the use of duplex ultrasound for a guided puncture. This might help to identify an appropriate puncture site and to reduce the number of misplaced punctures (including venous punctures). Before placing the closure device an angiography of the puncture site should be performed after the administration of vasodilators (e.g., nitroglycerin or verapamil) if possible. The inner balloon of the MYNXGRIP should be filled with diluted contrast-dye and the placement should be performed under radiographic control in order to ensure appropriate placement of the inner closure balloon. If the MYNX sealant protrudes close or even outside the skin level it is recommended to moisten it once with water or saline to prevent local skin irritations or even infections.

## 4. Summary

The safe and successful use of the MYNXGRIP closure device after repeated puncture of the brachial artery could be demonstrated in this case.
